# Email Consultations Between Patients and Doctors in Primary Care: Content Analysis

**DOI:** 10.2196/18218

**Published:** 2020-11-09

**Authors:** Helen Atherton, Anne-Marie Boylan, Abi Eccles, Joanna Fleming, Clare R Goyder, Rebecca L Morris

**Affiliations:** 1 Warwick Medical School University of Warwick Coventry United Kingdom; 2 Nuffield Department of Primary Care Health Sciences University of Oxford Oxford United Kingdom; 3 NIHR Greater Manchester Patient Safety Translational Research Centre University of Manchester Manchester United Kingdom

**Keywords:** remote consultation, communication, primary care

## Abstract

**Background:**

Increasingly, consultations in health care settings are conducted remotely using a range of communication technologies. Email allows for 2-way text-based communication, occurring asynchronously. Studies have explored the content and nature of email consultations to understand the use, structure, and function of email consultations. Most previous content analyses of email consultations in primary care settings have been conducted in North America, and these have shown that concerns and assumptions about how email consultations work have not been realized. There has not been a UK-based content analysis of email consultations.

**Objective:**

This study aims to explore and delineate the content of consultations conducted via email in English general practice by conducting a content analysis of email consultations between general practitioners (GPs) and patients.

**Methods:**

We conducted a content analysis of anonymized email consultations between GPs and patients in 2 general practices in the United Kingdom. We examined the descriptive elements of the correspondence to ascertain when the emails were sent, the number of emails in an email consultation, and the nature of the content. We used a normative approach to analyze the content of the email consultations to explore the use and function of email consultation.

**Results:**

We obtained 100 email consultations from 85 patients, which totaled 262 individual emails. Most email users were older than 40 years, and over half of the users were male. The email consultations were mostly short and completed in a few days. Emails were mostly sent and received during the day. The emails were mostly clinical in content rather than administrative and covered a wide range of clinical presentations. There were 3 key themes to the use and function of the email consultations: the role of the GP and email consultation, the transactional nature of an email consultation, and the operationalization of an email consultation.

**Conclusions:**

Most cases where emails are used to have a consultation with a patient in general practice have a shorter consultation, are clinical in nature, and are resolved quickly. GPs approach email consultations using key elements similar to that of the face-to-face consultation; however, using email consultations has the potential to alter the role of the GP, leading them to engage in more administrative tasks than usual. Email consultations were not a replacement for face-to-face consultations.

## Introduction

### Background

Increasingly, consultations in health care settings are conducted remotely using a range of communication technologies. Email allows for a 2-way text-based communication, occurring asynchronously. In general practice settings internationally, there has been varied adoption of email for consultation with patients. In Denmark, it is mandatory for all general practices to offer email consultations to their patients [1]. In the Netherlands, a survey demonstrated that 52.8% of practices offered email consultations [2]. Other countries have a less organized approach to the use of email for consultation. In the United Kingdom, just 6% of general practices reported offering email consultations to all patients; however, 21% of individual general practitioners (GPs) reported using email to have a consultation with a patient [3], reflecting the unstructured nature of this phenomenon in the UK general practice. Overall, email for consultation is not yet an embedded approach to having a consultation with a patient in general practice; a 2018 survey across 27 European countries reported that less than a fifth (19%) of GPs used email routinely to interact with patients about health-related issues [4].

Research has demonstrated that in the United Kingdom, where email consultation is not a mandatory offering and is not supported by 2-way secure encrypted email, email consultation is offered by GPs to selected patients and for selected problems, with GPs providing their own email addresses (National Health Service [NHS] or other addresses) to patients who are deemed to use this communication channel sensibly, not abusing the direct line to the doctor [5-7]. The selective approach to offering email interaction may reflect the concerns that GPs have about providing routine email consultations and the lack of protocol for processing email consultations [6].

These concerns include medico-legal implications and clinical safety issues about digital exclusion (eg, older patients, patients with low literacy) and increased workload via multiple contacts from and lengthy email exchanges with patients. UK general practice is funded by capitation; therefore, GPs are reimbursed not for individual consultations but according to patient numbers, regardless of the number of consultations conducted [1,6,8]. The potential for inequality is likely to be exacerbated when GPs are selective about who engages in email consultations. Despite this, the benefits of using email consultations have been described. Some patients appreciate the convenience and perceive improved access [1,5], and studies have shown that both patients and GPs feel that it promotes and sustains the doctor-patient relationship [1,6,8]. The written nature of the communication is seen as positive [6].

Quantitative evidence about the impact of email consultations on GPs’ workload, safety, and legal risks is inconclusive [9,10]. Studies have explored the content and nature of email consultations to understand whether concerns and benefits may be realized. Most previous content analyses of email consultations in primary care settings have been conducted in the United States and have found no evidence to support clinicians’ worries about receiving inappropriate and excessive numbers of emails [11-13], patients tended to use email for clinical rather than administrative enquiries [12,14], and emails often contained one or more problems [15]; however, it should be noted that in the United States, patients pay per consultation. Emails were most likely to be requests from patients, most commonly for medications or treatments [12,16]. An analysis of the content of email consultations in general practice in the Netherlands showed that this type of consultation was used for clinical issues, most popularly for psychological issues; when specific diagnoses were examined, the most common use of email consultation was for hypertension and diabetes [2].

There are numerous differences between the US and UK health systems, and to date, there has not been a content analysis of emails in UK general practice. Given the informal and unstructured approach to email consultations taken by GPs working in the United Kingdom [6], exploring the content of email consultations offers a way to examine how this type of consultation works where it is being used in practice and to see whether it leads to extended email exchanges and inappropriate content or safety issues in these instances.

### Objectives

We aim to explore and delineate the content of selected consultations conducted via email in English general practice by conducting the first content analysis of email consultations between GPs and patients in a UK setting.

## Methods

We sought to conduct a content analysis of email consultations between GPs and patients. Content analysis seeks to code, categorize, describe, and examine patterns in textual data. Content analysis takes an inductive approach to exploring data, not seeking to produce representative or generalizable findings but instead to identify concepts and patterns in the data and to produce numeric representations of various phenomena within the data.

The research team comprised 6 researchers. Each researcher has experience in mixed methods, qualitative research, and all work in primary care research; one of the researchers is a GP. The chief investigator has expertise in research on the use of email for consultation in general practice.

### Setting

The study was conducted in 2 general practices in the South-Central region of England, United Kingdom.

### Sampling of Participants

We purposively sampled 2 general practices for the use of email consultation. Participating practices had to have GPs who were using email to consult with patients. When this study was conducted, usage of email consultation was very low in the UK general practice, with just 6% of general practices offering it and 21% of GPs using it [3]. This limited the available types and numbers of practices that we could recruit from.

The 2 practices were selected such that they were different from each other with regard to the level of deprivation (measured using the Index of Multiple Deprivation 2015 [17]), practice size (the number of patients registered with the practice and the number of doctors), and the number of GPs within the practice who used email to consult with patients.

We selected 2 GPs to participate in the study, one from each practice. We collected information on the age, gender, and year of qualification of the participating GPs to inform the findings.

### Sampling of Emails

The emails adhered to a previously published and applied definition of an email consultation: a consultation intended to be a 2-way clinical communication between a GP and patient that was initiated by either party [6]. The content analysis was conducted on fully anonymized emails.

We used the number of email consultations collected in a previous primary care–based content analysis, which included 2 primary care professionals, as a guide (81 emails) [14] and aimed to collect 100 email consultations in total, with the option of obtaining more if needed as data analysis progressed. We continued the analysis until no new codes or themes occurred within the data set.

### Data Collection

Data collection was retrospective. Data collection occurred from February 01, 2017. Once recruited into the study, the participating GPs were asked to identify their last 50 email consultations (comprising all emails in the consultation) and make those available to the research team.

We collected fully anonymized email consultations from each participating GP, with both direct and quasi identifiers removed. We asked the participating GPs to retain the time and date that the email was sent so that we could look at the time at which the emails were sent and/or received, the number of emails in the consultation, and over what time period this occurred. Although emails were anonymized, we asked the participating GPs to withhold any emails that contained sensitive information or content and to note down if this happened.

Participating GPs gave written informed consent to participate in the study. Informed consent from patients was not required to use anonymized data for research purposes; however, before the data were anonymized and passed onto us, each patient was emailed by the GP to inform them about the study and to give them the option to opt out of having their emails in the study. No patients opted out, and 2 sent messages of support.

Procedures were put in place to ensure the safe transmission of anonymized data. A member of the participating general practice staff anonymized the content, which included removing direct identifiers (personal names, email addresses, and telephone numbers) and quasi identifiers (ethnicity, gender, date of birth, postcode, and location information). The messages were transferred electronically using a secure NHS email address to a researcher who is a practicing GP and holds a secure NHS email account. The GP author rechecked that the data were fully anonymized before sending them to the chief investigator. Any attachments to the emails were removed, but GPs were asked to note down if there had been any attachments so that we could present these data.

We also collected contextual data to provide background to the sample; both participating GPs noted the age and gender of the patients from each email. These aggregate data were sent separately and were not linked to the email content.

We used a strict protocol in the event of poor care or illegal activity being exposed within any of the emails.

### Ethical and Research Governance Approvals

Ethical approval was obtained from the University of Warwick Biomedical and Scientific Research Ethics Committee (reference number REGO-2016-1807), and research governance approval was granted by the UK NHS Health Research Authority.

### Data Analysis

We used a parallel design approach to our content analysis, using both descriptive and normative approaches [18].

The descriptive approach allowed us to identify the visible and obvious components of the data and to examine the frequency of their occurrence. We used this approach to examine each email for the following elements:

- The number of patients and their aggregated characteristics
- The number of emails in each consultation exchange, the time it took to respond to each email, and the period between the initial and final emails
- The time of the day emails were sent and responded to, both by patients and GPs
- The nature of the email content (eg, administrative and/or clinical content)
- Use of attachments (eg, images, audio files, or other supplements)

To give an overview of the breadth of content across the data set, we categorized the email content by presenting condition, and this allowed for emails where there was more than one condition discussed.

These descriptive data provided context for the normative approach to analysis. A normative approach involves the development of themes, in this case, to explore the use and function of email consultation. In this part of the analysis, the frequency of occurrence of data is not important, and a single occurrence of data could comprise a code.

For the normative approach, we produced an initial coding frame devised by the chief investigator and another researcher. Initially, this involved both researchers reading each email, making notes, and meeting to discuss these. We then examined the coding frameworks used in previous content analyses in health care settings [11,12,19] and the existing qualitative literature on the use of email consultation [20], the delivery of the consultation in general practice settings [21], and patient safety to develop the coding frame further by looking for overlaps [22].

The chief investigator and another researcher then formally coded every piece of data independently using our finalized coding frame, with the researchers then meeting to discuss where codes had been changed, merged, or added as data were examined again. Our patient safety researcher and practicing GP/researcher then read all the data and checked a sample of the coding samples to look for anything that could have been missed or that should have been added to the coding frame. Furthermore, 2 authors entered the coded data into NVivo (QSR International) qualitative analysis software, which was used to sort the data (see Multimedia Appendix 1 for a copy of the final coding frame).

In line with our normative approach, we developed high-level themes that categorized the content of the emails. We did this using conceptual mapping, adding each code to a visual map by hand and observing where they linked, until we identified recurring themes. The 2 researchers leading the analysis conducted this process together. However, throughout the interpretation stage, we held several meetings to ensure parity across the team; to enhance the validity of the analysis, we ensured that as a team, we agreed on the themes and their content. We were asked by the research ethics committee to use paraphrased examples from emails to illustrate the findings rather than direct quotations to ensure anonymity was maintained.

## Results

We obtained 100 email consultations that occurred with 85 individual patients. From practice 1, we obtained 50 email consultations with 45 patients, and from practice 2, 50 email consultations with 40 patients. The email consultations comprised 262 emails in total. None of the participating GPs reported editing or removing any email consultation because they deemed that the content was too sensitive to share.

### Participants

The participating practices were both in semirural (having both rural and urban characteristics) locations. Although they both had different deprivation scores, they were still in the least deprived deciles. Practice 1 had a smaller number of registered patients and 5 doctors, whereas practice 2 was much larger. In practice 1, just 1 GP offered email consultations to patients. In practice 2, there were 3 GPs offering email consultations. One GP from each practice participated in the study. Both participating GPs were male and had been using email consultation for 5 years with selected patients and offering patients only informal guidance on how to use it. Table 1 provides the details of the participating practices and the 2 participating GPs. Across both practices, 56% (48/85) patients engaged in the email consultations were male, and 39% (33/85) of the email consultations were with those in the age group of 40 to 59 years, with most patients being older than 40 years. When analyzed by practice, those who engaged in email consultations in practice 1 were older than those in practice 2. See Table 2 for a detailed breakdown of email user characteristics.

**Table 1:** Characteristics of participating practices and general practitioners.

Characteristics		Practice 1		Practice 2	
**Practice characteristics**					
	Location		Semirural		Semirural
	Deprivation score (1=most deprived and 10=least deprived)		10		7
	Number of patients registered at the general practice (as indication of practice size), n^a^		5000		14,000
	Doctors in participating practice, n		5		10
	GPs^b^ using email consultation within the practice, n		1		3
**GP characteristics**					
	Gender		Male		Male
	Age (years)		35		39
	Period for which the participating GP had been providing email consultation (years)		5		5
	Offering guidance to patients on how to use email consultation		Informal only, from GP		Informal only, from GP

^a^Value rounded to ensure anonymity of practice maintained.

^b^GP: general practitioner.

**Table 2:** Characteristics of patients and/or caregivers engaging in email consultations.

Characteristics		Whole sample (n=85), n (%)	Practice 1 (n=45), n (%)	Practice 2 (n=40), n (%)
**Sex**				
	Male	48 (56)	25 (56)	23 (57)
	Female	37 (44)	20 (44)	17 (43)
**Age (years)**				
	16-20	6 (7)	5 (11)	1 (2)
	20-29	4 (5)	2 (4)	2 (4)
	30-39	9 (11)	1(2)	8 (20)
	40-49	19 (22)	8 (18)	11 (28)
	50-59	14 (16)	9 (20)	5 (13)
	60-69	12 (14)	5 (11)	7 (18)
	70-79	16 (19)	11 (24)	5 (13)
	80-89	3 (4)	2 (5)	1 (2)
	≥90	2 (2)	2 (5)	0 (0)

### Handling of Emails

Both GPs approached email consultation in different ways, choosing to process and store them in different ways, though with the same outcomes, that the emails were recorded in the patient record as a consultation that had occurred.

### Email Consultations

There were 100 email consultations, with a total of 262 emails. Although we asked for email consultations that were 2-way, 15 email consultations that were sent to us comprised 1 email. Furthermore, 10 of those comprised just an email from the patient to the GP. We do not know if these were responded to via telephonic or face-to-face conversation; it is only known that there was no email response from the GP. Overall, 5 of the 1 way emails were from the GP to the patient. These 5 emails involved the transfer of information from the GP to patient, for example, a link to a website discussed during a face-to-face consultation, and there was no expected response.

Email consultations ranged from 1 email to a maximum of 9 emails in the conversation. The median number of emails in a consultation was 2. The number of days from the first email to the last email in the consultation ranged from 1 day to 1689 days. This was because 1 email consultation was reinitiated after a gap of 4 years, 7 months, and 15 days. With this anomaly excluded, the range is from 1 day to 351 days. The median number of days from the first to the last email was 3. GPs’ response times ranged from <1 day to 35 days, and the median time taken by GPs to respond was 2 days.

We were able to obtain the timestamp for 164 of the 262 emails; for those without a timestamp, the corresponding information was not present in the data transferred to us by the GPs. Of these, most emails were sent between 6 AM and 6 PM. Overall, 3 emails were sent after midnight and before 6 AM, and these were all sent by the GP from practice 2. More emails were sent by the GPs (18 emails) in the evening than by patients (7 emails). See Table 3 for full details.

**Table 3:** Time of the day when the emails were sent.

Time period	Sent by general practitioners (n=118), n (%)	Sent by patients (n=144), n (%)
Overnight (12 AM-6 AM)	3 (2.5)	0 (0)
Morning (6 AM-12 PM)	28 (23.7)	43 (29.9)
Afternoon (12 PM-6 PM)	31 (26.2)	34 (23.6)
Evening (6 PM-12 AM)	18 (15.3)	7 (4.9)
Not recorded	38 (32.2)	60 (41.7)
Total	118	144

### Content of the Email

Emails were used to discuss a wide range of complaints. Most email consultations contained clinical information (93/100); among these, several included some administrative content also (48/100). Very few email consultations were for administrative purposes only, with no clinical content (7/100). See Table 4 for the range of presentations discussed during an email consultation. These were not mutually exclusive, and more than one presentation appeared in many email consultations.

A total of 13 email consultations included an attachment. Overall, 9 of the attachments were photos, 2 were documents containing monitoring data (blood sugar levels and blood pressure), 1 was a fact sheet, and 1 was a form. In 11 email consultations, the contact was from carers on behalf of patients, 4 of which were about children and 7 about adults. In 13 emails, the GPs directly requested the patients to come in for a face-to-face consultation rather than continue the email consultation.

**Table 4:** Presentations discussed during email consultations.

Presentation	Examples of what was discussed
Cardiovascular	Blood pressure readings
Sexual health and family planning	Erectile dysfunction and contraception
Dermatology	Eczema
Gastroenterology	Constipation
Ear, nose, and throat	Labyrinthitis
Neurology	Brain injury
Orthopedic	Planned surgery
Palliative care	Relative in palliative care
Metabolic	Diabetes
Urological	Varicocele
Infectious disease	Lyme disease
Immunology	Allergic reaction
Preventive health	Vaccination
Medication	Side effects
Symptoms and advice	Fatigue
Administrative	Request for repeat prescription

### Use and Function of Email Consultation

Within our data set, we identified 3 overarching themes: the role of the GP and email consultation, the transactional nature of email consultation, and the operationalization of email as a mode of consultation.

#### Theme 1: The Role of the GP and Email Consultation

Email consultations did not always follow the traditional model of communication used in general practice. As email offered a direct line to the GP, patients could use email consultations to circumvent the receptionist who would normally act as gatekeeper to appointment booking and conducting administrative processes:

Yes, they are due the vaccinations, book the appointment, and I’ll order the vaccine.

GP’s response to patient’s email

Do nurses at the practice do cholesterol tests?

Patient’s enquiry to GP

This took the GP into an administrative role, or they directed the patient back to the reception staff. Sometimes, email consultations provided a *halfway*, with the patients asking whether they needed a face-to-face consultation for their particular presentation or providing information ahead of a consultation. Normally, patients might not be able to reach the GP to ask these questions directly:

I am sorry to hear you are in pain. Let’s discuss at the appointment you have booked in for Tuesday.

GP’s response to patient’s email

Patients were looking for clinical rather than administrative (receptionist) input on whether they needed a face-to-face consultation. This was time-saving for the patient but caused additional work for the GP, including undertaking the work of booking appointments:

I can see you tomorrow between 10 and 12 if convenient?

GP’s response to patient’s email

This also applied to cases where an email consultation led to a face-to-face consultation; the GP made an appointment, which took them outside of their role but saved time and effort for the patient. Sometimes it was the GP who made contact with the patient, offering reminders and scheduling care, indicating that for the GP, it was a choice to take on these tasks:

I think we planned to redo your bloods around now, shall I book you in?

GP’s email to patient

#### Theme 2: Transactional Nature of Email Consultation

In our data set, patients were largely responsible for initiating an email consultation. This mirrors how patients normally seek consultations, with most emails being initiated by patients. Multiple email consultations were initiated from the same patients within the data set, and there was an example of a patient *picking up* and restarting an email conversation after a long period.

Emails functioned as a way for the patients to request an action, for example, a change in a prescription or to ask directly for a test result, circumventing the administrative processes already in place to allow patients to request these particular actions from a GP:

Please can you alter my dose to be lower for the next fortnight?

Patient’s request to GP

I was wondering about the results of the blood test I had in March?

Patient’s request to GP

Where GPs initiated emails, it was for obtaining and/or providing information following up from a face-to-face consultation, for example, sending a link to a website on contraception choices or updates:

If you let me know how the cream goes, I can always ask for a specialist opinion.

GP’s email to patient

Email consultation was not only a way to contact patients but was also used to devolve responsibility back to the patients. There was an example of a GP using email to send information to patients, sending it to the patient via email during a face-to-face consultation and ready for the patient to use right after the consultation. In one email, a GP referred to having tried to call the patient and using email as the secondary option, as it was not possible to reach the patient via telephone, thus leaving the responsibility for the contact with the patient.

#### Theme 3: Operationalization of Email as a Mode of Consultation

The style of writing differed between GPs and among patients. Across emails from both GPs and patients, the salutation “Dear” was most commonly used but valedictions included “Best wishes,” “Many thanks,” and “Kind regards.” One GP always signed off an email with their first name, the other as “Dr [surname].” One GP was demonstrably less formal than the other, and patients were less formal in response.

The function of the email consultations was for health-related communication between a patient and their health care professional, in this case, a GP. However, some emails included non–health-related communication of a personal nature alongside this, for example, a patient thanking a GP for their help with a communication issue:

Thank you for taking the time to help me last Friday, I really appreciated you contacting the specialist.

Patient’s email to GP

The GPs applied some of the behaviors recognized as being key elements of general practice consultation and often employed several of these within one consultation. We observed signposting, negotiating with, and diagnosing the patient:

Please can you book in with the nurse for that.

GP signposting the patient

I would be happy to refer you, but I don’t think it is necessary at this point in time.

GP negotiating with the patient

I think the likely cause of your symptoms is an infection.

GP diagnosing patient

The most common behaviors were to engage with the patient and to apply safety netting:

Many thanks for this information, it is enough for me to make a decision.

GP engages with the patient

Please come back and see me in a month, but if you have any issues before, then please get back in touch.

GP using safety netting

Some consultations reverted to face-to-face consultations. An implicit working threshold was reached in the communication that led to the GP asking the patient to attend a face-to-face consultation. This was often where some form of physical and/or visual contact was required:

If you don’t mind coming in so I can have a look.

GP reverting to face-to-face consultation

Beyond clinical reasons for reverting to a face-to-face consultation, there were examples of boundary setting in relation to email consultation, whereby one lengthy email consultation culminated in the GP asking the patient to make an appointment rather than continue the conversation via email:

I think we need to book a telephone call please.

GP reverting to telephone consultation

Potential explicit safety issues were linked to the medium; for example, a delayed response could be an issue for urgent enquiries. As email consultations are remote and asynchronous, there is potential for emails to be missed or not read, without this delay in communication being immediately apparent to the patient or GP. There were 4 examples of this within the data, which led to a delayed response from GPs:

This email went to my junk folder, apologies.

Example of potential safety issue

I was on leave, so I have only just seen your email.

Example of potential safety issues

We only observed potential safety issues and not any actual safety incidents.

## Discussion

### Principal Findings

Most patients who were engaged in email consultations with the participating GPs were aged over 40 years, and over half of the users were male. The email consultations were mostly short and completed in a few days. There were exceptions, with one long and detailed email conversation noted. Emails were mostly sent and received during the day, although GPs did send some responses in the evening, outside of their working hours. Emails were mostly clinical in content rather than administrative and covered a wide range of clinical presentations. Of the 100 email consultations, 13 led to face-to-face consultations and 13 included an attachment. The styles of writing differed between the 2 participating GPs, with one being more formal than the other. This reflects the wide variation among GPs in their style of working and may influence how the correspondence is received and understood by patients. The email consultations had many of the same characteristics as face-to-face consultations. GPs deployed a range of actions including signposting, negotiating, and engaging with the patient.

### Limitations

There are limitations to our study, and the findings should be considered in light of these limitations. The participating GPs selected the email consultations sent to us, and so we may have ended up with a different range of emails than if we set up a study which collected email consultations as they happened. However, several included consultations that were long or critical of the GP, and there were no email consultations excluded because of their sensitive content. We received the emails once they had been selected and anonymized; therefore, there may be details or nuances that were missing as a result, for example, missing timings. However, as the data were collected retrospectively, there was no opportunity for the Hawthorne effect [23] to influence the behavior of either GP or patient in conducting the email consultation.

Our sample was limited in size, and the participating GPs were male and recently qualified, working in areas of low deprivation. This was because of the low levels of email consultation provided when the study was conducted [3]. We had taken an exploratory approach to data analysis and did not intend to extrapolate our findings across the entirety of general practice. Future studies could sample more widely and select doctors and practices from a wide range of areas. We do not have detailed information about the patients. We do not know how many other types of consultations they had with the GP. We do not know how long they had been using email to consult with the GP nor how it was initiated. Therefore, this analysis is conducted without these contexts, which could have influenced the depth of interpretation of the data.

Outside of a formalized email consultation system, emails were harder to obtain and required extensive data protection approaches. Systems using portals allow for more readily extracted data [16]. They also include a broader patient population than where email is used informally with selected patients, and we acknowledge that our sample limits the findings.

### Comparison With Prior Work

This is the first UK content analysis of email consultations, and our findings match closely with previous content analyses conducted in primary care settings in different countries. The patterns of usage that we observed in this study, with email consultations most likely to be requests from patients and concerning clinical rather than administrative contents, have also been demonstrated in previous content analyses in the United States [11-14,19]. We found that most email consultations were short, not sensitive in content, and did not include urgent content; these characteristics have also been demonstrated in previous content analyses carried out in countries other than the United Kingdom [11,14,19]. These commonalities are useful in determining the applicability of such studies about email consultation to the UK health system, indicating that there are lessons that the United Kingdom can learn from studies conducted in other countries. This is important given that email consultation use is likely to increase in the United Kingdom.

We identified a range of presentations by patients across the email consultations, with patients using email to consult about a wide range of issues, most commonly regarding medications and treatments. This matches the findings of other content analyses that looked at reasons for presentation using samples of email consultations [2,12,15,16,19]. Beyond content analyses, 2 studies set in the UK general practice have quantitatively examined the reasons that patients gave when leaving a web-based request for an appointment with a GP, known as an *online consultation* and acting as the first email in a consultation [24,25]. The reasons given closely mirror the presentations identified in our study, with administrative requests and medication enquiries featuring alongside a wide range of conditions. This is interesting, as our study did not seek to quantify or provide representative data but had similar findings to studies that used quantitative methodologies. Our findings were also well aligned with those identified in a study that looked at reasons for engaging in face-to-face consultations [26]. This may indicate that the written nature of an email consultation does not necessarily affect what people consult about as compared with a face-to-face consultation; however, further research is needed to confirm this.

The themes identified in our analysis of the content of email consultations were also similar to themes identified in qualitative research studies focused on the use of email consultation. Our study was not an in-depth qualitative exploration and therefore did not yield the same amount of data at the same depth. However, here we note that our themes can be seen in other studies. A qualitative interview study with GPs and patients about email consultation usage in the UK general practice found that patients liked to use email consultation as an alternative form of access to the GP, bypassing the reception staff [6]. Our study examined the text within an email consultation, and within the text, we saw that the role of the GP changed as a result of patients using email consultation. In the same qualitative interview study, both patients and GPs performed boundary setting in their use of email consultation, and we observed this in text-based interactions, classifying this as the operationalization of email consultation [6]. In a different qualitative interview study of patient and GP perspectives of email consultation in Denmark [1], it was found that patients valued the information transfer capability of email, and we observed emails being used for this purpose. This triangulation of findings indicates that future studies could benefit from combining both text-based analysis and in-depth qualitative approaches. Beyond the methodological implications, it indicates that examining the text of email consultations may be a way for health care services to understand how email consultation is functioning.

### Conclusions

In most instances where email is used to have a consultation with a patient in general practice, we observed that the consultation was short, clinical in nature, and resolved quickly. The GPs approached email consultation using an approach similar to that of face-to-face consultations; however, using email consultation had the potential to alter the role of the GP, leading them to engage in more administrative tasks than usual. Email consultation was not a replacement for face-to-face consultation for these GPs, but provided another way for the GPs and patients to engage with each other. On the basis of our analysis of their content, this form of consultation has the potential to provide an additional form of access for patients and to allow GPs to deal with certain issues quickly and without a face-to-face consultation.

## Appendix

Multimedia Appendix 1 Coding frame.

## Abbreviations

GP: general practitioner
NHS: National Health Service
