# Supplementary material for: Email Consultations Between Patients and Doctors in Primary Care: Content Analysis
Source: J Med Internet Res. 2020 Nov 9;22(11):e18218. doi: 10.2196/18218 (PMC7683246; doi:10.2196/18218)
Supplement: Multimedia Appendix 1 [file jmir_v22i11e18218_app1.docx]

**Patients**

**Requests for information about**

- Symptoms, problems, or diseases
- Psychosocial problems
- Tests or diagnostic procedures
- Referrals
- Medications or treatments
- Prevention

**Provision of information about**

- Symptoms, problems, or diseases
- Psychosocial problems
- Tests or diagnostic procedures
- Referrals
- Medications or treatments
- Prevention
- Extraneous info/feedback/other info (including, e.g. lifestyle info)

**Request for action**

- Physical examination
- Laboratory tests, x-rays, or other studies
- Test results
- Referral to other healthcare professional(s)
- Referral to non-h/c prof(s)
- Medications or treatments
- Appointment
- Information or advice
- Fit note
- Forms

**GP**

**GP initiates email**

- Advises action by patient
- Offers action (procedure/appointment/vaccine)
- Invites for appointment
- Provides info
- Requests info

**GP response to patient request:**

- Engages
- Fulfils
- Negotiates
- Reverts to face to face [examination, discussion, not specified]
- Reverts to telephone
- SUGGESTS action by patient
- Offers reassurance
- Offers referral
- Request for further information
- Information (e.g. about condition)
- Diagnosis
- Medications
- Safety-netting (if getting worse come back, etc.)
- Signposting (sending in right direction but taking them away from GP)

**Salutation**

- Title
- Name
- None

**Valediction**

- Formal
- Informal
